# Bearing Fault Diagnosis via Improved One-Dimensional Multi-Scale Dilated CNN

**DOI:** 10.3390/s21217319

**Published:** 2021-11-03

**Authors:** Jiajun He, Ping Wu, Yizhi Tong, Xujie Zhang, Meizhen Lei, Jinfeng Gao

**Affiliations:** School of Mechanical Engineering and Automation, Zhejiang Sci-Tech University, Hangzhou 310018, China; 201930507004@mails.zstu.edu.cn (J.H.); 201930507011@mails.zstu.edu.cn (Y.T.); 201920505009@mails.zstu.edu.cn (X.Z.); leimeizhen@zstu.edu.cn (M.L.); jfgao@zstu.edu.cn (J.G.)

**Keywords:** multi-scale, CNN, dilated convolutional, fault diagnosis

## Abstract

Bearings are the key and important components of rotating machinery. Effective bearing fault diagnosis can ensure operation safety and reduce maintenance costs. This paper aims to develop a novel bearing fault diagnosis method via an improved multi-scale convolutional neural network (IMSCNN). In traditional convolutional neural network (CNN), a fixed convolutional kernel is often employed in the convolutional layer. Thus, informative features can not be fully extracted for fault diagnosis. In the proposed IMSCNN, a 1D dimensional convolutional layer is used to mitigate the effect of noise contained in vibration signals. Then, four dilated convolutional kernels with different dilation rates are integrated to extract multi-scale features through the inception structure. Experimental results from the popular CWRU and PU datasets show the superiority of the proposed method by comparison with other related methods.

## 1. Introduction

Bearings are regarded as critical components in rotating machinery. However, bearings often suffer from failure conditions, since they are usually working in a harsh working environment [1,2]. Early and effective bearing fault diagnosis technique plays an important role in avoiding unforeseen downtime of rotating machinery.

Compared to current signals [3] and acoustic emission signals [4], vibration signals [5,6] contain abundant information that reflects the health state of bearings. Thus, vibration signals are widely used in bearing fault diagnosis. Generally, fault diagnosis techniques can be categorized into two types, signal analysis, and data-driven methods. For signal analysis methods, vibration signals are first dealt with signal processing methods such as time-domain analysis [7], frequency domain analysis [8] and time-frequency domain analysis [9,10]. Then, based on the expert knowledge, features extracted from different domains are used to detect bearings health changes and assess health states. A major limitation of signal analysis methods is that comprehensive and great expert knowledge is required to determine the health states and faulty types of bearings from extracted features.

Different from signal analysis methods, data-driven methods only rely on the collected vibration data for fault diagnosis. In data-driven methods, labeled vibration data are first collected. Then, features are extracted from different domains similar to the signal analysis method. For further fault diagnosis purpose, with these extracted features, classifiers are trained using machine learning methods such as Support Vector Machine (SVM) [11,12], Random Forest (RF) [13,14] and Multi-Layer Perceptron (MLP) [15].

Recently, deep learning methods have gained considerable attention in the field of data-driven fault diagnosis. A huge advantage of deep learning is that deep features can be extracted automatically. Generally, deep features can exhibit more useful information for fault diagnosis, compared to shallow features extracted from traditional machine learning methods. It has proved that better diagnostic performance can be achieved using deep learning methods [16]. As representative deep learning methods, auto-encoder (AE), deep belief networks(DBN), and convolutional neural network (CNN) have shown their superiority in bearing fault diagnosis. For example, Chen and Li fed the extracted time-domain and frequency-domain features from the different sensor signals into multiple two-layer sparse autoencoders (SAE) neural networks for fault classification [17]. Gan et al. designed a two-layer hierarchical diagnosis network (HDN) to identify fault types and recognize fault severity ranking by employing deep belief networks (DBNs) to provide representative features [18].

As one of the most effective deep learning methods, a convolutional neural network (CNN) has also been applied to fault diagnosis. The common CNN-based methods can be categorized into one-dimensional (1-D) CNN-based and two-dimension (2-D) CNN-based methods. For 1-D CNN methods, the raw 1-D time-domain vibration signals are directly fed into the 1D CNN model [19]. For 2-D CNN methods, the raw vibration signals are usually transformed into 2-D time-frequency domain data, and then the 2D data are dealt with 2D-CNN [20]. Levent et al. [19] took the raw vibration data as the input, and used the compact adaptive 1D-CNN to diagnose the bearing fault. Gao et al. [21] proposed a novel hybrid deep learning method (NHDLM) based on Extended Deep Convolutional Neural Networks with Wide First-layer Kernels (EWDCNN) and long short-term memory (LSTM) to enhance diagnosis accuracy for rotating machinery in complex environments. Han et al. [22] presented a novel diagnosis framework that combines the Spatio-temporal pattern network (STPN) approach with CNN and applied it to fault diagnosis of complex systems. Wang et al. [23] fused the multi-sensor vibration signals and transformed them into images to obtain more informative features. Then the input was fed to the bottleneck CNN for fault diagnosis. However, in CNN-based fault diagnosis methods, each convolutional operation often uses convolutional kernels of the same size. To further extract more informative features from vibration data, inspired by the inception structure, convolutional kernels of different sizes can be selected to improve the performance of fault diagnosis. To extract multi-scale features, Qiao et al. [24] employed the convolutional kernels with different widths to act as filters with different scales of frequency domain resolution to simultaneously extract features of different frequency bands of the vibration signal. Further, Wang et al. [25] combined the dilated convolutional with multi-scale convolutional for remaining useful life prediction. Compared with the convolutional layer, dilated convolutional layer has a larger receptive field with the same size convolutional kernel. Due to this advantage, dilated convolutional can ignore the redundant information in vibration signals.

Motivated by the above discussions, an improved multi-scale convolutional neural network is developed for bearing fault diagnosis in this paper. To extract more informative features, we employ four dilated convolutional kernels with different dilation rates in multi-scale CNN. Among these four dilated convolutional kernels, the dilation rates of the two kernels are set as 1. Thus, these two dilated convolutional kernels become convolutional kernels. Moreover, different from multi-scale CNN [24], a 1D convolutional layer is adopted before using multi-scale CNN to mitigate the effect of noise for bearing fault diagnosis. In summary, the main contributions of the proposed method can be listed as follows,

(1) To enlarge the receptive of multi-scale CNN, four dilated convolutional kernels with different dilation rates are designed. Thus, more informative features can be extracted for fault diagnosis.
(2) For reduction of the noise in vibration signals, an additional one-dimensional convolutional layer is adopted to extract the features before dilated convolutional layer.
(3) Two widely used datasets including CWRU and PU datasets are employed to evaluate the performance of the proposed method compared with other related methods. Results show the superiority of the proposed method.

The rest of this paper is organized as follows. Section 2 offers a brief review of CNN and its inception structure. In Section 3, the improved multi-scale dilated CNN is developed for bearing fault diagnosis. Two widely used experimental cases are carried out to evaluate the performance of the proposed method compared with other related methods in Section 4. In the final section, the conclusions are drawn.

## 2. Related Works

### 2.1. Convolutional Neural Networks (CNN)

CNN is one of the most popular deep neural networks in recent years [26]. It has been widely used in computer vision [27], natural language processing [28] and other fields [29]. CNN usually consists of three parts: (1) convolutional layer; (2) pooling layer; (3) fully connected (FC) layer.

#### 2.1.1. Convolutional Layer

Due to the characteristics of sparse interactions and parameter sharing of convolutional operation, the number of weights of convolutional operation is determined by the size and number of the convolutional kernel in CNN. Each convolutional kernel deals with a part of the input data. Thus, the number of weights is significantly reduced. Meanwhile, the convolutional operation has the characteristics of equal representations since the fixed convolutional kernel is adopted. The convolutional operation is below,

(1) $$x_{j}^{l}=f\left(\sum_{i\in M_{j}}x_{i}^{l-1}*k_{j}^{l}+b_{l}^{i}\right)$$

where $x_{j}^{l}$ represents the *j*th output of the *l*th layer network. $M_{j}$ is the number of inputs. $k_{j}^{l}$, $b_{j}^{l}$ and ∗ represent the convolutional kernel, the bias and the convolutional operation, respectively. f(·) is the activation function.

Compared to the commonly used sigmoid function, ReLU function has become a ubiquitous activation function in DNN, due to its computational efficiency and the ability of reducing the gradient vanishing. ReLU function is defined as follows,

(2) $$Z_{j}^{l}=f\left(x_{j}^{l}\right)=\max\left\{0,x_{j}^{l}\right\}$$

where $x_{j}^{l}$ is the input and $Z_{j}^{l}$ is the output.

#### 2.1.2. Pooling Layer

To reduce the overfitting, pooling layers are often adopted in CNN. The commonly used pooling methods include average pooling and max pooling. In this study, the max pooling is adopted.It can be defined below:

(3) $$P_{j,t}^{l}=\max W_{t}\left(Z_{j}^{l}\right)$$

where $P_{j,t}^{l}$ is the *t*th output in the *j*th feature map of the *l*th layer network. $W_{t}\left(\cdot \right)$ represents the *t*th sliding window operation for input with the size of n×n pooling window.

#### 2.1.3. Fully Connected Layer

For classification, a fully connected (FC) layer is usually employed as classifier where all the inputs from one layer are connected to every activation unit of the next layer. The formula of FC layer is defined as follows:

(4) $$x_{j}^{l}=f\left(\sum_{i=0}^{m-1}x_{i}^{l-1}\times k_{ij}^{l}+b_{l}^{i}\right)$$

where $x_{j}^{l}$ is the output of the *l*th FC layer and $x_{i}^{l-1}$ is the output of l−1th layer.

### 2.2. Dilated Convolutional Neural Networks

Although the pooling layer is widely used to maintain invariance and control over-fitting, it will suffer from the reduction of spatial resolution. Thus, the spatial information of the feature map would be lost. The dilated convolutional layer was developed in the field of image segmentation to address this issue [30]. Dilated convolutional can expand the receptive field without increasing the number of parameters or the amount of calculation. The formula for dilated convolutional is as follows:

(5) $$x_{j}^{l}=f\left(\sum_{i\in M_{j}}D_{r}\left(x_{i}^{l-1}\right)*k_{ij}^{l}+b_{j}^{i}\right)$$

where $D_{r}$ represents the dilated operation with dilation rate *r*.

**Figure 1:**
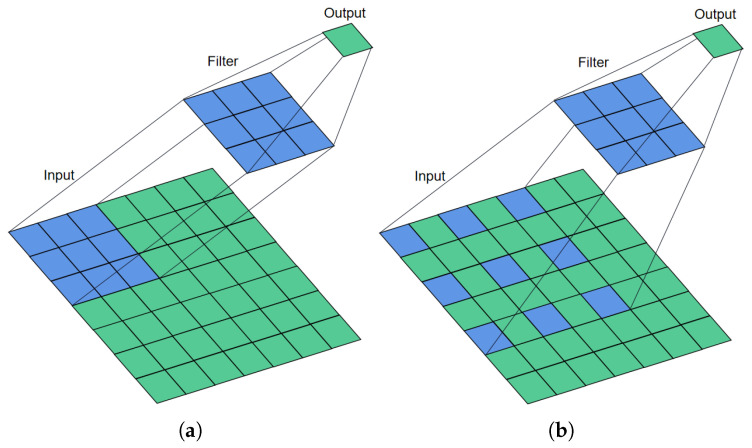
The calculation process of dilated convolutional (r=1,2). (**a**) Dilated convolutional with dilation rate 1 (Traditional convolution); (**b**) Dilated convolutional with dilation rate 2.

In dilated convolutional operation, dilation rate defines a spacing between the values in a kernel. Figure 1 shows the 3 × 3 convolutional process with dilation rates of 1 and 2, respectively. As displayed in Figure 1, if the dilation rate is set to 1, the dilated convolutional becomes the traditional convolutional. In Figure 1b, a receptive field of 5 × 5 is presented (r=1), while a receptive field of 3 × 3 is obtained (r=2) as shown in Figure 1a.

### 2.3. Inception Architecture

**Figure 2:**
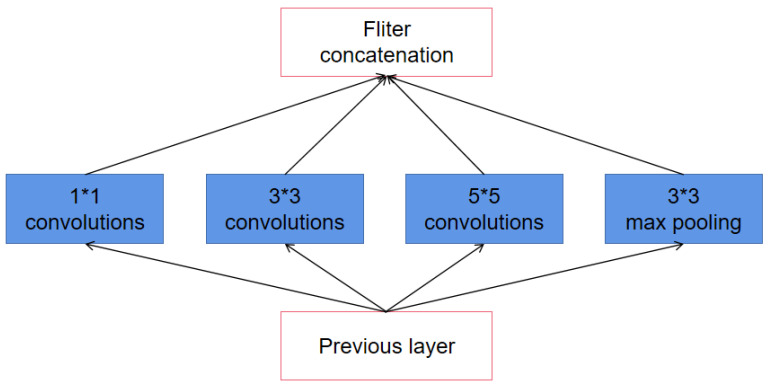
Diagram of the inception structure V1.

To improve the performance of networks, inception architecture was introduced in CNN. In GoogLeNet [31], the inception V1 structure was developed as shown in Figure 2. By employing the concept of inception architecture, the depth and width of the networks are increased. Additionally, the advantage of inception V1 architecture is that computational costs can be reduced.

As shown in Figure 2, there are three convolutional kernels with different sizes and a max-pooling layer in inception V1 architecture. The formula of inception can be expressed as:

(6) $$F^{l}=\left[F_{C1}^{l};F_{C2}^{l};F_{C3}^{l};F_{P}^{l}\right]$$

where $F_{C1}^{l}$, $F_{C2}^{l}$, $F_{C3}^{l}$ and $F_{P}^{l}$ represent feature maps after three convolutional layers with different convolutional kernel sizes and pooling layer, respectively. $F^{l}$ represents the feature map of *l*th layer which combines $F_{C1}^{l}$, $F_{C2}^{l}$, $F_{C3}^{l}$ and $F_{P}^{l}$.

Since $F^{l}$ contains features computed over different scales, the subsequent network will select the more useful features adaptive.

## 3. The Architecture of the Proposed IMSCNN

In the proposed method, the raw vibration data is used as the input of the neural network. The raw vibration data is divided into a number of groups. To facilitate subsequent processing and accelerate the convergence of the neural networks, maximum-minimum normalization is used to deal with each group of input data,

(7) $$in_{i}^{\mathrm{normalize}\;}=\frac{in_{i}-in^{\min}}{in^{\max}-in^{\min}},\;i=1,2,\ldots ,N$$

where $in_{i}$ is the *i*th sample. $in_{i}^{\min}$ and $in_{i}^{\max}$ are the smallest and largest values in the group. *N* is the size of samples in group.

**Figure 3:**
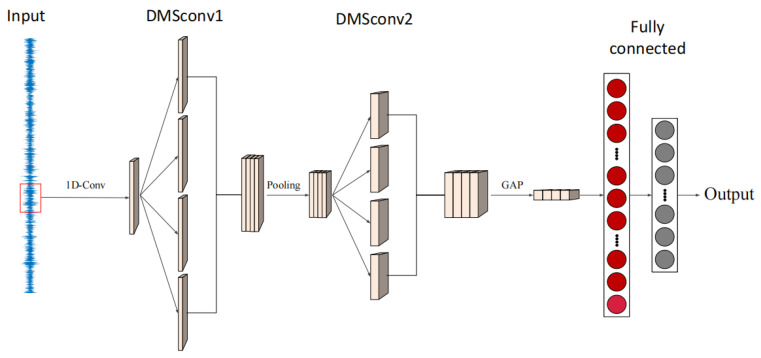
Structure of the proposed IMSCNN method.

**Figure 4:**
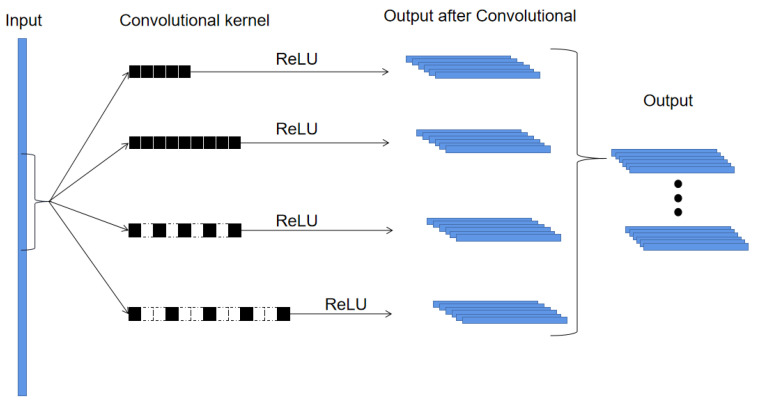
Structure of DMSConv layer.

The structure of the proposed IMSCNN is shown in Figure 3. In practice, the vibration signals are often contaminated by noises. To alleviate this problem, a 1-D convolutional layer is first employed in the proposed method. By using a 1D convolutional layer, noises contained in the raw vibration signals can be filtered. To enhance the ability of feature extraction, a dilated multi-scale convolutional (DMSConv) layer with a larger kernel size is employed to extract multi-scale features. Inside the DMSConv layer, there are four multi-scale convolutional as shown in Figure 4.

In the DMSconv layer, four dilated convolutional kernels with different dilation rates are integrated to extract features through the inception structure. The details of DMSconv layer are shown in Table 1, where KS and NC represent the kernel size and number of channels, respectively. From Table 1, it is noticed that the kernel size of each convolutional layer is singular in multi-scale convolutional. This is for the convenience of using the same convolutional to unify the size of feature map output. Therefore, the number of output channels of the DMSconv layer is 4 × NC.

The multi-scale feature map (MSFM) is defined as,

(8) $$\mathrm{MSFM}=\left[F_{T1};F_{T2};F_{D1};F_{D2}\right]$$

where the $F_{T}$ and $F_{D}$ represent the feature maps after dilated convolutions, respectively.

To increase the robustness and reduce the computational effort, the max-pooling operation is performed on the FM obtained after the first DMSconv layer. To extract deeper features, a second DMSconv layer with a smaller kernel size is utilized. Additionally, the global average pool (GAP) is used to compress the features of each channel into four features. Finally, these features are fed into an FC layer for classification.

The structure of the proposed IMSCNN model is shown in Table 2. Usually, vibration signals are often collected under high-frequency noise background. Thus, in the first and second DMSconv layers, relatively large kernel size and small kernel size are selected to suppress the high-frequency noise. According to [32], the kernel sizes of the two DMSconv layers are 32 and 2 in this study, respectively. To train the proposed IMSCNN model, cross-entropy loss function is adopted for fault diagnosis.

The widely used Adam [33] method is employed. And batch normalization (BN) [34] is used to regularize the model and reduce the need for Dropout,

(9) $${\hat{x}}^{i}=\frac{x^{i}-E\left(x^{i}\right)}{\sqrt{\mathrm{Var}\left(x^{i}\right)}}$$

where $x^{i}$ represents the output of the *i*th layer.

## 4. Experiments and Results

To verify the performance of the proposed IMSCNN method, two cases including CWRU and PU datasets are carried out. For comparison, the widely used neural networks including MLP, CNN and MSCNN are employed. The details of these neural networks are described as follows,

- MLP: it is composed of five FC layers. The details are shown in the Table 3.
- CNN: it is composed of four one-dimensional convolutional pooling layers (Conv&Pool) and three FC layers. The activation function is ReLU. The details are shown in the Table 4.
- MSCNN: its main structure is the same as IMSCNN, where the dilation rate is set as 1.
- SimpleIMSCNN: The structure of SimpleIMCNN is similar to IMSCNN, except the first 1D convolutional layer shown in Figure 3 is ignored.

**Table 3:** Architecture-related hyperparameters of MLP.

NO.	Layer Name	Layer Size
1	FC1	1024×1
2	FC2	512×1
3	FC3	256×1
4	FC4	128×1
5	FC5	64×1
6	FC6	output×1

**Table 4:** Architecture-related hyperparameters of CNN.

NO.	Layer Name	Layer Size
1	Conv&Pool1	15×1×16
2	Conv&Pool2	3×1×32
3	Conv&Pool3	3×1×64
4	Conv&Pool4	3×1×128
5	GAP	4
6	FC1	512×1
7	FC2	256×1
8	FC3	64×1
9	FC4	output×1

For all comparative methods, the batch size is set to 64. Adam is used as an optimizer. The maximum number of epochs is selected as 100. 1024 data points are set as a group of data input to the neural network. The working environment is Intel Core i7-8750h CPU@ 2.20 GHz, 24.0 GB ram, and Geforce GTX 2070 GPU under Windows 10 operating system. All methods are implemented through Python 3.6.12 and Pytorch 1.7.1.

### 4.1. Case 1: CWRU

The CWRU datasets were provided by the Case Western Reserve University bearing data center [35]. The vibration data was collected under three faulty conditions and one normal condition. Each fault has three kinds of faults in different positions, so there are a total of 9 kinds of faults to be classified. In this study, the data with the acquisition frequency of 12 K is selected. The details of the fault are shown in the Table 5. Table 5 shows that in addition to the normal bearings there are three different fault locations, Ball (B), Inner Race (IR), and Out Race (OR). Each fault location contains three fault diameters of 0.07 inches, 0.014 inches, and 0.021 inches respectively. All faults were created manually by electro-discharge machining (EDM).

**Figure 5:**
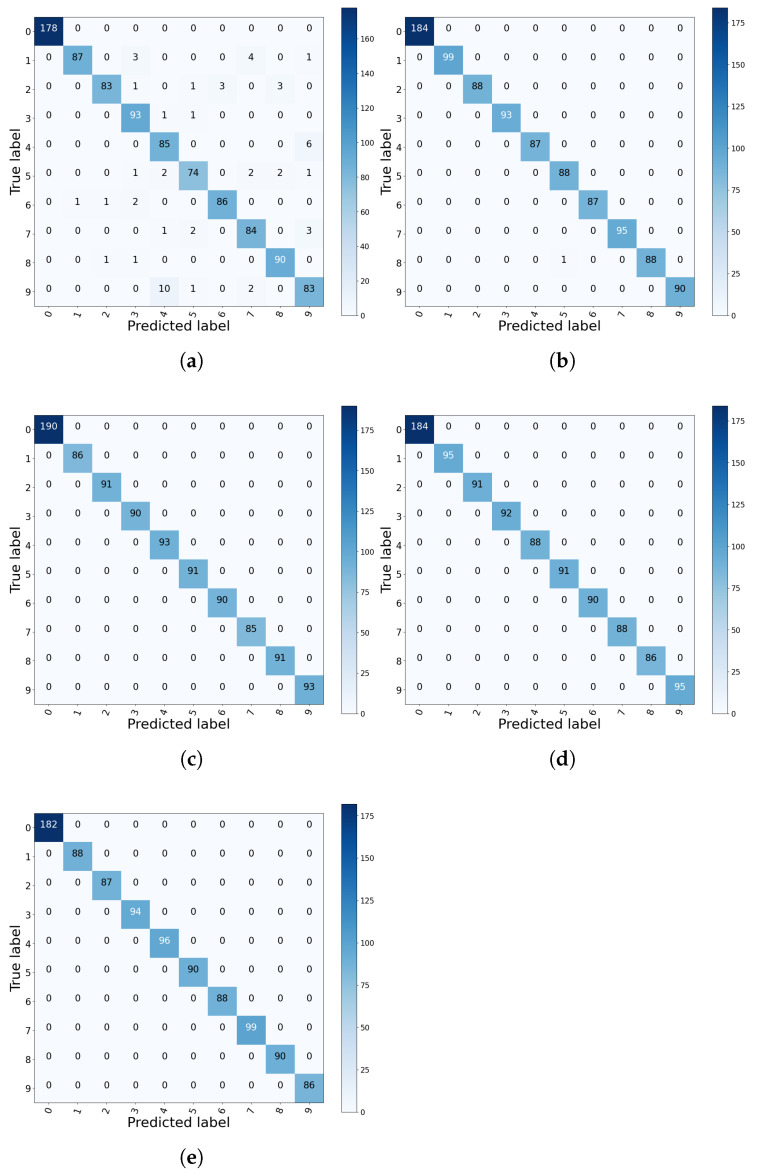
Confusion matrix of the CWRU dataset. (**a**) MLP; (**b**) CNN; (**c**) SimpleIMSCNN; (**d**) MSCNN; (**e**) IMSCNN.

In the experiment, 80% of the collected data from each condition is used for training and the other 20% is for testing. The accuracy results are shown in Table 6. The confusion matrix is shown in Figure 5. It can be seen from Table 6 that both CNN and MLP offer satisfactory performance, where the accuracy reaches 99.77% and 94.63% respectively. Through extracting multi-scale features, MSCNN, SimpleIMSCNN, and IMSCNN can provide 100% accuracy.

**Figure 6:**
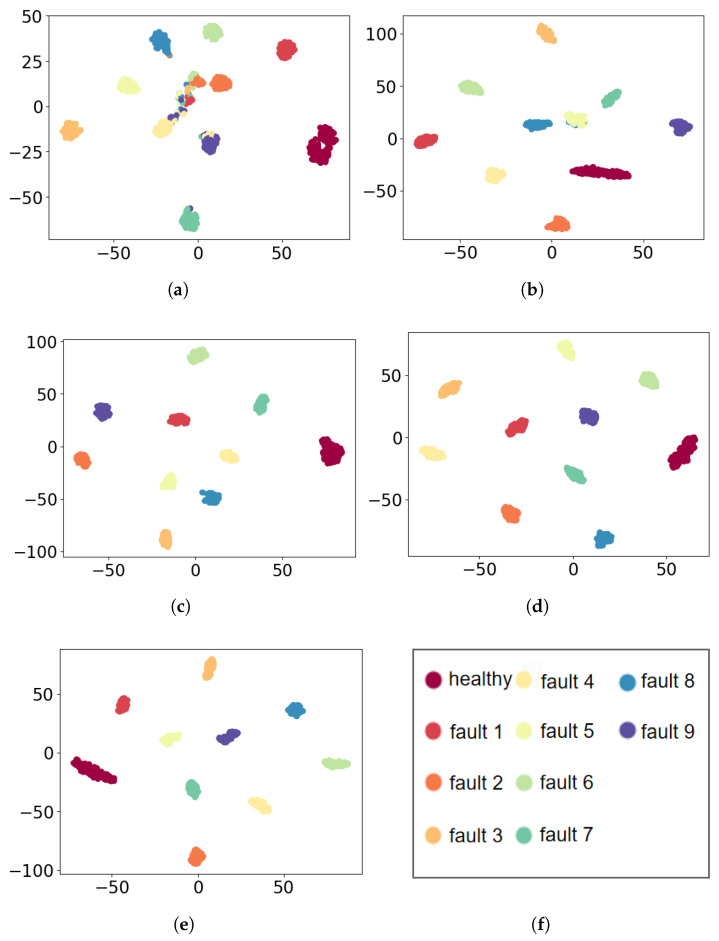
Feature visualization via t-SNE: CWRU dataset. (**a**) MLP; (**b**) CNN; (**c**) SimpleIMSCNN; (**d**); MSCNN (**e**) IMSCNN (**f**) class of the each color.

To further compare the ability of feature extraction, t-SNE [36] is used to visualize the extracted features for all methods. As shown in Figure 6, it can be found that the features extracted by MLP are close between class 1, class 4, class 7, and class 8, while the features extracted by CNN are close between classes 5 and 8. Thus, there exist misclassified results by MLP and CNN. The data in the confusion matrix can also prove this point as plotted in Figure 5. Contrary, the distance between features extracted from MSCNN, SimpleIMSCNN, and IMSCNN are relative far.

### 4.2. Case 2: PU Dataset

PU datasets were provided by the Paderborn University Bearing Data Center [37]. In the PU dataset, there are 14 faulty conditions. In this study, the vibration data was collected under the working conditions of rotating speed 1500 rpm, load torque 0.7 nm, and radial force 1000 N. The descriptions of 14 faults are listed in Table 7. In Table 7, the fault location is represented by fault mode. Since the fault type of NO.13 KI04 is the same as NO. 8 KI14, we only consider NO. 8 KI14. Thus, our goal is to classify the 13 faulty conditions.

All data are collected on the test rig through the transducer. The sampling frequency of vibration data is 64 k Hz and the sampling time is 4 s. The real damages bearing used in this experiment were obtained by accelerated lifetime test. Low viscosity oil was also used during the experiments, which was more conducive to the appearance of damage. Most damage is caused by fatigue damages, which arise in the form of pittings. The rest of the damage types are mainly plastic deformation in the form of indentations caused by the debris. We use 80% of the data from each condition for training and 20% for testing.

**Figure 7:**
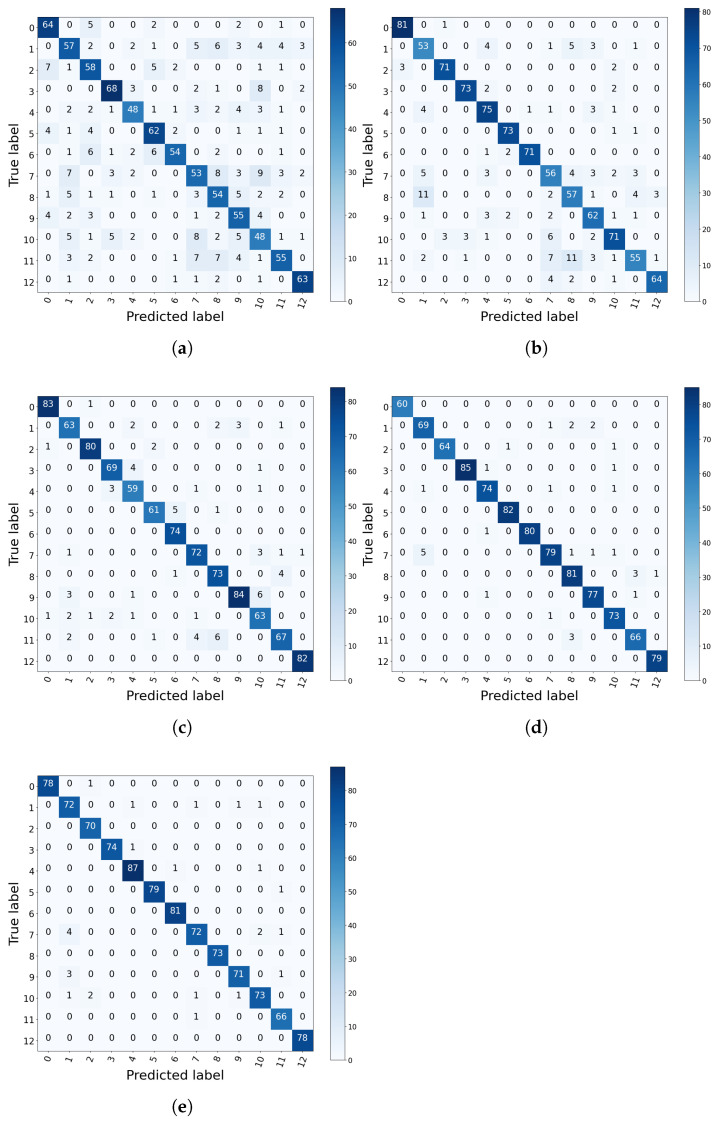
Confusion matrix of the result on each method with PU dataset. (**a**) MLP; (**b**) CNN; (**c**) SimpleIMSCNN; (**d**) MSCNN (**e**); IMSCNN.

The confusion matrix is displayed in Figure 7. As shown in Figure 7, there are many faulty samples misclassified by MLP and CNN. For MLP, the accuracy rate is only 58.89% for fault 7. For CNN, the accuracy of fault 11 is only 67.9%, and 13.58% of samples of fault 11 are misclassified as fault 8. From the data in confusion matrices of SimpleIMSCNN, MSCNN, and IMSCNN, it can be seen that there are much fewer misclassified samples.

**Figure 8:**
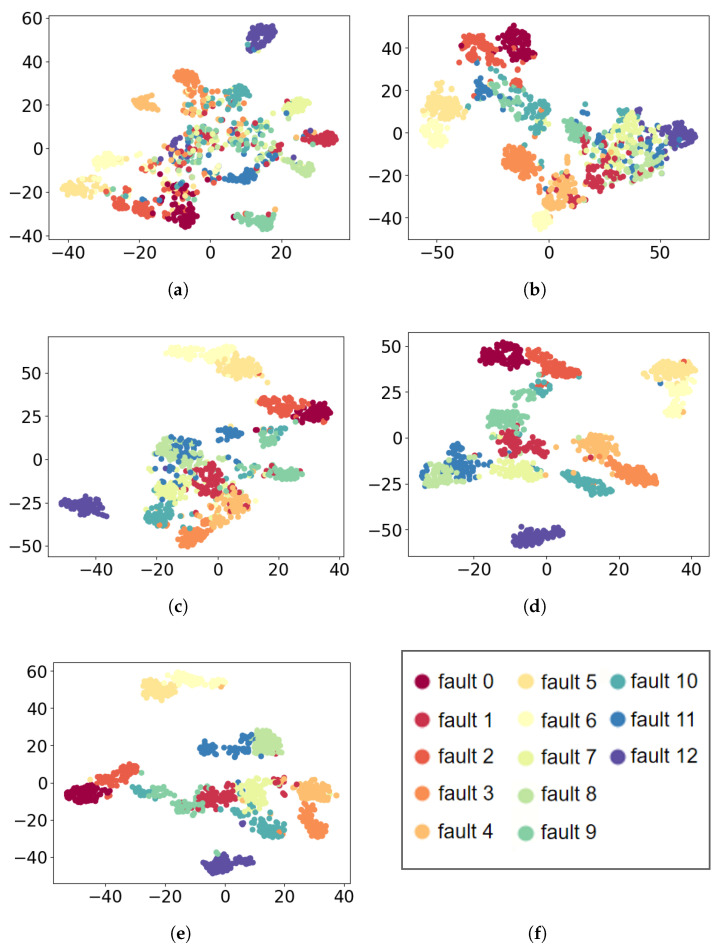
Feature visualization via t-SNE: PU dataset. (**a**) MLP; (**b**) CNN; (**c**) SimpleIMSCNN; (**d**) MSCNN; (**e**) IMSCNN; (**f**) class of the each color.

In a similar way, t-SNE is used to visualize the extracted for all comparative methods. The visualization results are plotted in Figure 8. As displayed in Figure 8, it can be seen that the features extracted from MLP and CNN can not be well separated. Compared to MSCNN and SimpleIMSCNN, the features extracted from IMSCNN are more distinguishable.

Table 8 lists the accuracy results. From Table 8, the accuracy of MLP and 1DCNN are 69.69% and 85.64%, respectively. Through extracting multi-scale features, the accuracy of MSCNN is 95.53%. On the other hand, the accuracy of SimpleIMSCNN is 92.1%. The proposed IMSCNN method can provide the best performance among the comparative methods, where the accuracy reaches 96.55%. It indicates that the noises contained in vibration signals can be filtered by the first 1D convolutional layer of the proposed IMSCNN. Thus, the diagnostic performance of IMSCNN is improved.

## 5. Conclusions

A novel CNN-based bearing fault diagnosis method called IMSCNN is developed in this paper. In the proposed IMSCNN method, the one-dimensional original vibration signal is preprocessed through a one-dimensional convolutional layer to alleviate the influence of noise. To extract more informative features, a multi-scale feature extraction layer called the DMSCov layer which consists of four dilated convolutional operation with different dilation rates is employed for fault diagnosis. Two widely used CWRU and PU datasets are utilized to verify the superiority of the proposed IMSCNN by comparison with other related methods.

## Figures and Tables

**Table 1:** Architecture-related hyperparameters of DMSconv layer.

NO.	Layer Name	Layer Size	Dilation Rate
1	MSConv1	(KS+1)×1×NC	1
2	MSConv2	(KS∗2+1)×1×NC	1
3	MSConv3	(KS+1)×1×NC	2
4	MSConv4	(KS+1)×1×NC	3

**Table 2:** Architecture-related hyperparameters of IMSCNN.

NO.	Layer Name	Layer Size
1	Conv	17×1×64
2	DMSconv1	KS = 32 NC = 32
3	Pool	2×1
4	DMSconv2	KS = 2 NC = 64
5	GAP	4
6	FC1	1024×1
7	FC2	64×1
8	FC3	output×1

**Table 5:** Description of each type of fault in the CWRU dataset.

NO.	Bearing State	Fault Diameters	Fault Location
0	Health	/	/
1	Fault 1	0.007 inch	IR
2	Fault 2	0.014 inch	IR
3	Fault 3	0.021 inch	IR
4	Fault 4	0.007 inch	B
5	Fault 5	0.014 inch	B
6	Fault 6	0.021 inch	B
7	Fault 7	0.007 inch	OR
8	Fault 8	0.014 inch	OR
9	Fault 9	0.021 inch	OR

All the vibration signals were collected under same motor loads at 1797 rpm and 0 HP.

**Table 6:** The accuracy of each method in CWRU dataset.

Method	Acc
MLP	94.63%
CNN	99.77%
MSCNN	100%
SimpleIMSCNN	100%
IMSCNN	100%

**Table 7:** Detailed description of PU datasets.

NO.	Bearing Code	Fault Mode	Description
0	KA04	Outer ring damage (SP, S, Level 1)	Caused by fatigue and pitting
1	KA15	Outer ring damage (SP, S, Level 1)	Caused by plastic deform and indentation
2	KA16	Outer ring damage (SP, R, Level 2)	Caused by fatigue and pitting
3	KA22	Outer ring damage (SP, S, Level 1)	Caused by fatigue and pitting
4	KA30	Outer ring damage (D, R, Level 1)	Caused by plastic deform and indentation
5	KB23	Outer ring and innerring damage (SP, M, Level 2)	Caused by fatigue and pitting
6	KB24	Outer ring and innerring damage (D, M, Level 3)	Caused by fatigue and pitting
7	KB27	Outer ring and innerring damage (D, M, Level 1)	Caused by plastic deform and indentation
8	KI14	Inner ring damage (SP, M, Level 1)	Caused by fatigue and pitting
9	KI16	Inner ring damage (SP, S, Level 1)	Caused by fatigue and pitting
10	KI17	Inner ring damage (SP, R, Level 3)	Caused by fatigue and pitting
11	KI18	Inner ring damage (SP, S, Level 1)	Caused by fatigue and pitting
12	KI21	Inner ring damage (SP, S, Level 2)	Caused by fatigue and pitting
13	KI04	Inner ring damage (SP, M, Level 1)	Caused by fatigue and pitting

[1] SP: single point fault; D: distributed fault. [2] S: single damage; R: repetitive damage; M: multiple damage.

**Table 8:** The accuracy of each method in PU data set.

Method	Acc
MLP	69.69%
CNN	85.64%
MSCNN	95.53%
SimpleIMSCNN	92.10%
IMSCNN	96.55%

## References

[1] Aiwina H., Sheng Z., Tan A.C., Mathew J. (2009). Rotating machinery prognostics: State of the art, challenges and opportunities. Mech. Syst. Signal Process..

[2] Qian Y., Yan R., Gao R.X. (2017). A multi-time scale approach to remaining useful life prediction in rolling bearing. Mech. Syst. Signal Process..

[3] Naha A., Samanta A.K., Routray A., Deb A.K. (2017). Low complexity motor current signature analysis using sub-Nyquist strategy with reduced data length. IEEE Trans. Instrum. Meas..

[4] Tomoya M., Katsumi I., Masana K. (1994). Acoustic emission during fatigue crack growth in carburized gear tooth. Trans. Jpn. Soc. Mech. Eng. Part C.

[5] Wang W., McFadden P. (1996). Application of wavelets to gearbox vibration signals for fault detection. J. Sound Vib..

[6] Luo G., Osypiw D., Irle M. (2000). Real-time condition monitoring by significant and natural frequencies analysis of vibration signal with wavelet filter and autocorrelation enhancement. J. Sound Vib..

[7] Long X., Yang P., Guo H., Zhao Z., Wu X. (2019). A CBA-KELM-based recognition method for fault diagnosis of wind turbines with time-domain analysis and multisensor data fusion. Shock Vib..

[8] Wang T., Qi J., Xu H., Wang Y., Liu L., Gao D. (2016). Fault diagnosis method based on FFT-RPCA-SVM for cascaded-multilevel inverter. ISA Trans..

[9] Shen Z., Chen X., Zhang X., He Z. (2012). A novel intelligent gear fault diagnosis model based on EMD and multi-class TSVM. Measurement.

[10] Gao T., Sheng W., Zhou M., Fang B., Luo F., Li J. (2020). Method for Fault Diagnosis of Temperature-Related MEMS Inertial Sensors by Combining Hilbert–Huang Transform and Deep Learning. Sensors.

[11] Yang Y., Yu D., Cheng J. (2007). A fault diagnosis approach for roller bearing based on IMF envelope spectrum and SVM. Measurement.

[12] Zhou S., Qian S., Chang W., Xiao Y., Cheng Y. (2018). A novel bearing multi-fault diagnosis approach based on weighted permutation entropy and an improved SVM ensemble classifier. Sensors.

[13] Li C., Sanchez R.V., Zurita G., Cerrada M., Cabrera D., Vásquez R.E. (2016). Gearbox fault diagnosis based on deep random forest fusion of acoustic and vibratory signals. Mech. Syst. Signal Process..

[14] Zhang Y., Luo L., Ji X., Dai Y. (2021). Improved Random Forest Algorithm Based on Decision Paths for Fault Diagnosis of Chemical Process with Incomplete Data. Sensors.

[15] Waqar T., Demetgul M. (2016). Thermal analysis MLP neural network based fault diagnosis on worm gears. Measurement.

[16] Jia F., Lei Y., Lin J., Zhou X., Lu N. (2016). Deep neural networks: A promising tool for fault characteristic mining and intelligent diagnosis of rotating machinery with massive data. Mech. Syst. Signal Proces..

[17] Chen Z., Li W. (2017). Multisensor Feature Fusion for Bearing Fault Diagnosis Using Sparse Autoencoder and Deep Belief Network. IEEE Trans. Instrum. Meas..

[18] Gan M., Wang C., Zhu C. (2016). Construction of hierarchical diagnosis network based on deep learning and its application in the fault pattern recognition of rolling element bearings. Mech. Syst. Signal Process.

[19] Eren L., Ince T., Kiranyaz S. (2019). A generic intelligent bearing fault diagnosis system using compact adaptive 1D CNN classifier. J. Signal Process. Syst..

[20] Yuan J., Han T., Tang J., AN L. (2017). An approach to intelligent fault diagnosis of rolling bearing using wavelet time-frequency representation and CNN. Mach. Des. Res..

[21] Gao Y., Kim C.H., Kim J.M. (2021). A Novel Hybrid Deep Learning Method for Fault Diagnosis of Rotating Machinery Based on Extended WDCNN and Long Short-Term Memory. Sensors.

[22] Han T., Liu C., Wu L., Sarkar S., Jiang D. (2019). An adaptive spatiotemporal feature learning approach for fault diagnosis in complex systems. Mech. Syst. Signal Process..

[23] Wang H., Li S., Song L., Cui L. (2019). A novel convolutional neural network based fault recognition method via image fusion of multi-vibration-signals. Comput. Ind..

[24] Qiao H., Wang T., Wang P., Zhang L., Xu M. (2019). An adaptive weighted multiscale convolutional neural network for rotating machinery fault diagnosis under variable operating conditions. IEEE Access.

[25] Wang R., Shi R., Hu X., Shen C. (2021). Remaining Useful Life Prediction of Rolling Bearings Based on Multiscale Convolutional Neural Network with Integrated Dilated Convolution Blocks. Shock Vib..

[26] LeCun Y., Boser B., Denker J., Henderson D., Howard R., Hubbard W., Jackel L. (1989). Handwritten digit recognition with a back-propagation network. Adv. Neural Inf. Process. Syst..

[27] Krizhevsky A., Sutskever I., Hinton G.E. (2012). Imagenet classification with deep convolutional neural networks. Adv. Neural Inf. Process. Syst..

[28] Dos Santos C., Gatti M. Deep convolutional neural networks for sentiment analysis of short texts. Proceedings of the COLING 2014, the 25th International Conference on Computational Linguistics: Technical Papers.

[29] Anwar S.M., Majid M., Qayyum A., Awais M., Alnowami M., Khan M.K. (2018). Medical image analysis using convolutional neural networks: A review. J. Med. Syst..

[30] Yu F., Koltun V. (2015). Multi-scale context aggregation by dilated convolutions. arXiv.

[31] Szegedy C., Liu W., Jia Y., Sermanet P., Reed S., Anguelov D., Erhan D., Vanhoucke V., Rabinovich A. Going deeper with convolutions. Proceedings of the IEEE Conference on Computer Vision and Pattern Recognition.

[32] Zhang W., Peng G., Li C., Chen Y., Zhang Z. (2017). A new deep learning model for fault diagnosis with good anti-noise and domain adaptation ability on raw vibration signals. Sensors.

[33] Kingma D.P., Ba J. (2014). Adam: A method for stochastic optimization. arXiv.

[34] Ioffe S., Szegedy C. Batch normalization: Accelerating deep network training by reducing internal covariate shift. Proceedings of the International Conference on Machine Learning.

[35] Case Western Reserve University Bearing Data Center Website. https://engineering.case.edu/bearingdatacenter.

[36] Van der Maaten L., Hinton G. (2008). Visualizing data using t-SNE. J. Mach. Learn. Res..

[37] Lessmeier C., Kimotho J.K., Zimmer D., Sextro W. Condition monitoring of bearing damage in electromechanical drive systems by using motor current signals of electric motors: A benchmark data set for data-driven classification. Proceedings of the European Conference of the Prognostics and Health Management Society.

